# Elevated myocardial extracellular volume fraction in Duchenne muscular dystrophy

**DOI:** 10.1186/1532-429X-18-S1-P243

**Published:** 2016-01-27

**Authors:** James Starc, Ryan A Moore, Chet Villa, John L Jefferies, Michael D Taylor

**Affiliations:** grid.239573.90000000090258099Cardiology, Cincinnati Children's Medical Center, Cincinnati, OH USA

## Background

Duchenne muscular dystrophy (DMD) is a genetic, X-linked recessive disease. The clinical course in DMD consists of progressive skeletal muscle wasting and weakness. Cardiac involvement in DMD is characterized by myocardial fibrosis leading to dilated cardiomyopathy, progressive heart failure, and arrhythmias. Earlier detection of cardiac involvement and appropriate treatment thereof holds potential to improve outcomes. Cardiovascular magnetic resonance (CMR) imaging for detecting disease progression in DMD patients is largely based on late gadolinium enhancement (LGE) imaging. LGE-CMR is clinically limited because it is by nature a late finding, unable to detect diffuse myocardial fibrosis. CMR extracellular volume (ECV) quantification using T1 mapping is a histologically validated, non-invasive marker of diffuse fibrosis. We hypothesized that ECV will be elevated in the DMD population and correlate with additional metrics of left ventricular function.

## Methods

We performed a retrospective review of pediatric DMD subjects who have undergone CMR at a single institution. A total of forty-seven DMD patients (mean age 14 ± 2 years) with LGE and ECV imaging were included for analysis.

## Results

Global myocardial ECV was significantly higher in the DMD group (29 ± 6%) compared with published normal values (24 ± 2%, p = 0.0001). Higher ECV values correlate with other indices of left ventricular function, including left ventricular ejection fraction (r=-0.46, p = 0.001) and indexed left ventricular end diastolic volume (r = 0.41, p =0.004). ECV did not significantly correlate with increasing age (r = 0.07, p = 0.065). ECV was not significantly higher in DMD patients with LGE (30 ± 7%) compared to DMD patients without LGE (27 ± 5%, p = 0.0717).

## Conclusions

CMR T1 mapping is a feasible method for quantification of ECV in patients with DMD. Global myocardial ECV is significantly higher in the DMD population compared to healthy controls and correlates with other metrics of myocardial function. Global myocardial ECV may serve as an important tool to determine cardiac involvement in DMD population and help guide medical management.

